# Integrative Omics Reveal Female‐Specific Benefits of p16^+^ Cell Clearance in Aging Mice

**DOI:** 10.1002/advs.202509444

**Published:** 2025-10-30

**Authors:** Yao Lin, Boshi Wang, Mengling Huang, Justina C. Wolters, Marco Demaria

**Affiliations:** ^1^ European Research Institute for the Biology of Ageing (ERIBA) University Medical Center Groningen (UMCG) University of Groningen (RUG) Groningen 9713AV The Netherlands; ^2^ Department of Pediatrics University Medical Center Groningen (UMCG) University of Groningen (RUG) Groningen 9713AV The Netherlands

**Keywords:** aging, cellular senescence, p16, SASP, senolytics

## Abstract

Aging is marked by the accumulation of cells expressing the cyclin‐dependent kinase inhibitor p16Ink4a. These p16⁺ cells, largely senescent, contribute to inflammation and tissue dysfunction. While eliminating p16⁺ cells improves healthspan, sex‐specific differences in their burden and clearance remain unclear. Through combined transcriptomic, proteomic, and functional analyses, we reveal distinct sex‐dependent dynamics of p16⁺ cells during aging. Female mice accumulate significantly more p16⁺ cells across multiple tissues, particularly in the liver. In the p16‐3MR model, selective ablation of these cells enhances grip strength, promotes skin regeneration, and reduces liver damage exclusively in females. Multi‐omics profiling shows that p16⁺ cell removal shifts female liver expression toward youthful, health‐associated profiles, marked by improved mitochondrial activity and reduced inflammatory signaling—molecular patterns resembling those induced by longevity interventions such as calorie restriction, rapamycin, and acarbose. Integrative analysis of our and independent datasets identifies a conserved transcriptional network involving Srm, Cd36, and Lrrfip1, suggesting shared mitochondrial–immune regulatory mechanisms. Overall, our findings establish p16⁺ cells as critical yet heterogeneous drivers of tissue aging, uncover sex‐specific differences in their abundance and senolytic responsiveness, and support the development of precision senotherapeutics that consider sex as a key biological variable in aging and rejuvenation.

## Graphical abstract

Aging causes accumulation of p16⁺ senescent cells that promote inflammation and dysfunction. Multi‐omics analyses reveal striking sex differences in senescent cell burden and clearance: females accumulate more p16⁺ cells, especially in the liver, and their removal rejuvenates molecular and functional profiles. Conserved mitochondrial–immune networks underlie these effects, underscoring sex as key for precision senotherapeutics.

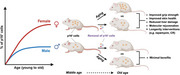

## 1 Introduction

Aging is a multifactorial process characterized by progressive physiological decline across multiple biological levels.^[^ ^1^ ^]^ At the organismal level, aging manifests as reduced strength and resilience, and an increased risk of morbidity and mortality.^[^ ^1^, ^2^ ^]^ Molecular and cellular damage progressively accumulates, accompanied by dysregulated transcriptomic and proteomic profiles, particularly affecting pathways involved in RNA biosynthesis, mitochondrial biogenesis, and inflammation, eventually leading to tissue dysfunction and reduced regenerative capacity.^[^ ^3^, ^4^ ^]^ Notably, age‐related changes exhibit sexual dimorphism, influencing lifespan and disease susceptibility differently between the sexes.^[^ ^5^ ^]^

The field of longevity intervention has advanced rapidly, with dietary and pharmacological strategies demonstrating efficacy in extending lifespan and healthspan.^[^ ^6^, ^7^, ^8^ ^]^ However, most interventions exhibit sex‐dependent effects.^[^ ^5^, ^9^, ^10^ ^]^ For example, calorie restriction (CR), which is known to extend lifespan and healthspan in both rodents and primates, has sex‐specific effects: it reduces fat mass more in young males than females in both mice and overweight or obese humans;^[^ ^11^ ^]^ it influences lifespan differently in male and female mice depending on genetic background;^[^ ^12^, ^13^ ^]^ and among non‐obese healthy adults, it more strongly improves risk factors for cardiovascular disease and type 2 diabetes in men than in women.^[^ ^14^ ^]^ Pharmacologically, acarbose treatment increases the median lifespan by 17% in males compared to 5% in females, with sex‐specific benefits: improved rotarod performance in females and enhanced glucose metabolism in males.^[^ ^15^, ^16^ ^]^ Rapamycin shows a stronger mortality reduction in females (HR = 0.41) than in males (HR = 0.63) across 29 studies.^[^ ^17^ ^]^ The variability in intervention efficacy between the sexes underscores the importance of evaluating longevity strategies separately in males and females. Despite the growing recognition of sex‐specific responses, the molecular and cellular mechanisms underlying these differences remain poorly understood. Identifying the key regulators of sexual dimorphism in aging and the responses to antiaging interventions is a critical unmet need, limiting the development of tailored and broadly effective therapies.

A key strategy for understanding the aging process and the effects of interventions involves genome‐wide omics technologies such as RNA sequencing (RNA‐Seq) and proteomics. For example, the Tabula Muris Consortium analyzed RNA‐Seq data from 17 organs across 10 time points to identify both shared and organ‐specific gene expression changes in males and females.^[^ ^18^ ^]^ Tyshkovskiy et al. conducted RNA‐Seq analyses of eight longevity interventions and later expanded their study to 17 interventions.^[^ ^19^ ^]^ At the protein level, aging and the impact of antiaging interventions have been systematically profiled to identify potential therapeutic targets.^[^ ^20^, ^21^ ^]^ These large‐scale transcriptomic and proteomic datasets not only deepen our understanding of aging biology, but also serve as valuable resources for evaluating sex‐specific responses to interventions.

Cellular senescence is characterized by a stable cell cycle arrest mediated through the upregulation of the cyclin‐dependent kinase inhibitors p21^WAF1/Cip1^ and p16^INK4A^ (encoded by *Cdkn1a* and *Cdkn2a*, respectively, in mice).^[^ ^3^, ^22^, ^23^ ^]^ While p16^INK4A^ expression is one of the most widely used biomarkers of senescence, it does not define the state exclusively. Indeed, p16⁺ cells represent a heterogeneous population, most of which are senescent, but some arise from alternative stress or differentiation programs.^[^ ^24^, ^25^ ^]^

With advancing age, p16⁺ cells accumulate in multiple tissues, where they are generally associated with tissue dysfunction, chronic inflammation, and various age‐related pathologies.^[^ ^26^ ^]^ For example, senescent hepatocytes display impaired fatty acid oxidation, contributing to hepatic lipid accumulation and correlating with the severity of nonalcoholic fatty liver disease (NAFLD).^[^ ^27^ ^]^ Targeting p16⁺ or senescent cells in the liver reduces steatosis, mitigates chemotherapy‐induced toxicity, and attenuates NAFLD progression in mice.^[^ ^27^, ^28^ ^]^

Preclinical studies have demonstrated that selective clearance of p16⁺ cells extends health span and lifespan while reversing age‐related dysfunction.^[^ ^24^, ^29^, ^30^, ^31^, ^32^ ^]^ However, not all p16⁺ cells are deleterious and, in certain contexts, they play beneficial or reparative roles. For instance, transient p16⁺ cells promote optimal wound healing in the skin^[^ ^25^ ^]^ and contribute to liver regeneration following injury.^[^ ^33^ ^]^ Moreover, specific immune populations, particularly macrophages, can exhibit high p16^INK4A^ expression without classical senescence features.^[^ ^34^, ^35^, ^36^ ^]^ Their elimination may disrupt tissue homeostasis or impair regenerative responses.

Thus, while the removal of p16⁺ cells generally confers health benefits, broad ablation strategies may also carry risks, emphasizing the need to define the cellular identity, context, and functional state of p16⁺ cells prior to intervention. In addition, emerging evidence suggests sex‐dependent differences in the accumulation of age‐associated of p16⁺ cells,^[^ ^37^ ^]^ yet the functional consequences of these differences and their impact on clearance outcomes remain poorly characterized.

In this study, we used publicly available datasets and a transgenic reporter model to examine the age‐related accumulation of p16⁺ cells and the effects of their selective clearance in both male and female mice.

## 2 Results

### 2.1 Differential Burden and Clearance Responses of p16⁺ Cells in Aging Male and Female Mice

**Figure 1:**
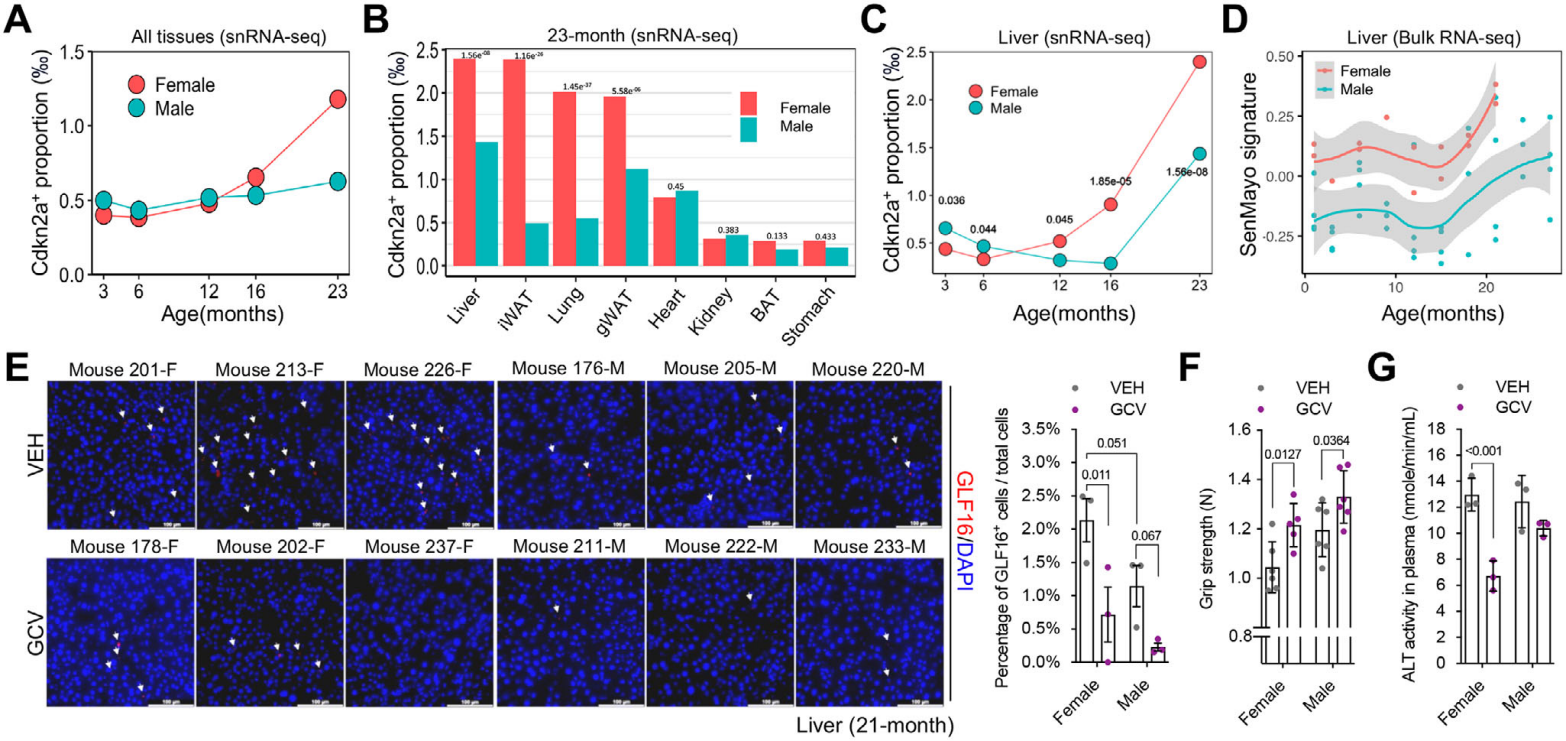
Differential burden and clearance responses of *Cdkn2a*⁺ cells in aging male and female mice. A) Proportions of *Cdkn2a*⁺ cells in wild‐type female and male C57BL/6 mice across five age groups (3, 6, 12, 16, and 23 months). The proportions were aggregated from eight core tissues: liver, kidney, heart, stomach, lung, brown adipose tissue (BAT), inguinal white adipose tissue (iWAT), and gonadal white adipose tissue (gWAT). Cells with nonzero *Cdkn2a* expression were classified as *Cdkn2a* ^+^ cells. Total cells analyzed per age group for females. *n* = 721919, 1232847, 1156100, 639357, 1309937. For males: *n* = 617989, 130691, 1243660, 626931, 1200416. B) Tissue‐ and sex‐specific proportions of *Cdkn2a*⁺ cells in 23‐month‐old mice. Significance was assessed using a two‐sided Pearson's chi‐squared test. Total cells analyzed per tissue for females (left to right): *n* = 150937, 88531, 222278, 99653, 208633, 291790, 154873, 93242. For males: *n* = 125421, 93105, 189061, 90758, 199121, 314457, 122636, 65857. C) Age‐ and sex‐dependent proportions of Cdkn2a⁺ cells in liver tissue. Significance was assessed using a two‐sided Pearson's chi‐squared test. Total cells analyzed per age group for females. *n* = 116634, 178409, 90474, 114022, 150937. For males: *n* = 102217, 207937, 96930, 52267, 125421. Analyses in panels (A)–(C) were based on the publicly available single‐nucleus RNA‐Seq dataset GSE247719 (*N* = 2 mice/group at 3 and 16 months; *N* = 4 mice/group at 6, 12, and 23 months). D) Age‐ and sex‐dependent senescence signature scores (SenMayo gene sets) from gene set variation analysis (GSVA) based on liver bulk RNA‐Seq GSE132040. Trend lines show LOESS smoothing (span = 0.75) with 95% confidence intervals. Each point represents a mouse. E) Representative images and quantification of GLF16 staining in the livers of 21‐month‐old VEH‐ or GCV‐treated p16‐3MR female and male mice (scale bar = 100 µm, *N* = 3 mice/group). F) Grip strength measurement in 24‐month‐old VEH‐ or GCV‐treated p16‐3MR female and male mice (*N* = 5 or 6 mice/group). G) Plasma alanine aminotransferase (ALT) activity in 21‐month‐old VEH‐ or GCV‐treated p16‐3MR female and male mice (*N* = 3 per group). For (E), (F), and (G), two‐way ANOVA (Fisher's LSD test), data are shown as the mean ± SD, and *p*‐values are labeled on the graphs. GCV, ganciclovir, was dissolved in pH11 water. VEH, vehicle (pH11 water).

p16 (encoded by *Cdkn2a*) is a key marker of senescent cells. First, we used a publicly available snRNA‐Seq dataset^[^ ^38^ ^]^ to characterize the sex‐specific temporal dynamics of the burden of *Cdkn2a^+^* senescent cells during natural aging. We included the liver, kidney, heart, stomach, lung, brown adipose tissue (BAT), inguinal white adipose tissue (iWAT), and gonadal white adipose tissue (gWAT). The overall proportion of *Cdkn2a^+^* cells (aggregated across all eight tissues) increased with age in both sexes (**Figure** **1**A). Nevertheless, females exhibited a more rapid accumulation than males between 16 and 23 months of age (Figure 1A). The odds of a cell being *Cdkn2a^+^* in females at 23 months were ≈3‐fold higher than that at 3 months, whereas this increase was 57% lower in males. At 23 months of age, both sexes showed the highest proportion of *Cdkn2a^+^* cells. Thus, we further examined the tissue‐specific proportions at this age and found that four of the eight tissues (liver, lung, iWAT, and gWAT) showed a significantly higher proportion of *Cdkn2a^+^* cells in females than in males (Figure 1B), whereas the remaining tissues exhibited no sex difference. As the liver is a key driver of sex differences in metabolism, we further investigated sex‐ and age‐dependent dynamics in the liver and confirmed that aged females accumulated more *Cdkn2a^+^* senescent cells (Figure 1C). To further dissect the observed increase in *Cdkn2a⁺* cells with age, we analyzed organ‐ and cell type–specific changes in *Cdkn2a⁺* cell proportions and overall cell‐type composition at 6 and 23 months in both sexes (Table S1, Supporting Information). In the female liver specifically, immune cell types, including lymphoid and myeloid cells, show both an intrinsically higher proportion of *Cdkn2a⁺* cells and increased abundance at 23 months compared to 6 months. In contrast, hepatocytes exhibit a higher proportion of *Cdkn2a*⁺ cells with age, despite a reduction in their overall abundance, possibly as a consequence of age‐associated changes in cell composition. These findings suggest that the age‐related increase in *Cdkn2a⁺* cells in female mice results from a combination of cell‐intrinsic upregulation and population‐level shifts in cell‐type composition. Since single‐nucleus RNA sequencing (snRNA‐Seq) captures only nuclear transcripts and may therefore provide an incomplete view of cellular transcriptomes, we complemented the nuclear transcriptomic analysis by evaluating the burden of senescent cells in the liver across sexes and ages using an independent bulk RNA‐Seq dataset,^[^ ^18^ ^]^ which includes both nuclear and cytoplasmic transcripts. Consistent with the snRNA‐Seq findings, the livers of aged females exhibited a higher score for the senescence‐gene set signature SenMayo^[^ ^39^ ^]^ (Figure 1D), indicating a higher number of senescent cells.

Given the sex‐dependent accumulation of *Cdkn2a^+^* cells during natural aging, particularly in the liver (Figure 1A–D), we sought to determine whether there was any sex‐specific differential response to p16^+^ cell clearance. For this, we used the p16‐3MR mouse model, in which a 3MR cassette was knocked into the p16^Ink4a^ locus within a BAC, such that its expression reflects transcriptional activation of p16. The HSV‐tk component of the 3MR transgene allows for selective elimination of p16^+^ cells through the administration of the prodrug ganciclovir (GCV).^[^ ^25^ ^]^ We treated middle‐aged male and female p16‐3MR mice (16‐18 months of age) with one round of vehicle (VEH) or GCV (five consecutive days) every month, followed by a series of analyses (Figure S1A, Supporting Information). qPCR analysis of p16 using selective primers confirmed increased expression in aged livers and lungs compared to the young controls (Figure S1B, Supporting Information). Aged females exhibited higher p16 relative expression level compared to young counterparts, and GCV treatment significantly reduced p16 expression (Figure S1B, Supporting Information). Using GLF16 fluorescence staining, a recently developed senescence reporter that detects lysosomal β‐galactosidase activity independent of pH,^[^ ^23^, ^40^ ^]^ we independently confirmed that the proportion of senescent cells was higher in aged female livers than in males, with a trend toward statistical significance (*p* = 0.051), and that GCV led to a more significant reduction in p16⁺ cells in females (Figure 1E and Figure S2A, Supporting Information). Furthermore, we stained for IL1α — a well‐established SASP factor and senescence marker — in aged livers and discovered that VEH‐treated aged females exhibited higher IL1α abundance compared to males. Importantly, GCV treatment significantly reduced IL1α levels (Figure S2B, Supporting Information), further supporting the efficacy of GCV treatments in clearing senescent cells. To assess the overall functional health and frailty in aged mice, we measured forelimb grip strength, a widely used surrogate for musculoskeletal integrity and physical performance. Clearance of p16⁺ senescent cells led to improved grip strength in both sexes, but the effect was significantly stronger in aged females, suggesting a sex‐specific advantage of senescent cell removal (Figure 1F).

In the skin, removal of p16⁺ cells during active wound healing can impair optimal tissue repair in both young and aged mice.^[^ ^24^, ^25^ ^]^ In contrast, the clearance of senescent cells that naturally accumulate with age has been shown to reduce the signs of aging and promote faster wound healing.^[^ ^41^ ^]^ Similarly, long‐term GCV‐treated aged females, but not males, exhibited a better‐preserved skin architecture with larger hair follicles, significantly thicker dermis and epidermis (Figure S3A, Supporting Information), and improved wound healing capacity (Figure S3B, Supporting Information). In the liver, senescent cells contribute to tissue dysfunction and elevated ALT levels, and their clearance reduces liver damage and improves tissue health. Consistent with reduced liver damage, GCV‐treated mice showed lower plasma ALT levels, with the reduction being significantly greater in females than in males, relative to their respective sex‐matched vehicle‐treated controls (Figure 1G). Taken together, these findings demonstrate that aged female mice show higher level of p16^+^ cells and derive greater functional benefits from their clearance, including increased grip strength, enhanced skin regeneration, and reduced liver damage.

### 2.2 Clearance of p16⁺ Cells in Aged Female Livers Recapitulates Effects of Antiaging Interventions

To identify the molecular mediators underlying the beneficial effects of p16⁺ cell clearance, we performed genome‐wide transcriptomic profiling of liver tissue from p16‐3MR mice treated with vehicle (VEH) or ganciclovir (GCV). Differential gene expression analysis was conducted and contextualized through integrative comparisons with publicly available RNA‐seq datasets representing natural aging and pharmacological or dietary antiaging interventions. For aging‐associated transcriptomic signatures, we utilized the Tabula Muris Senis dataset, which provides high‐resolution RNA‐seq data across 10 age points and for both sexes.^[^ ^18^ ^]^ To assess overlap with established longevity paradigms, we incorporated data from Tyshkovskiy et al.,^[^ ^19^ ^]^ which included RNA‐seq profiles from male and female mice subjected to calorie restriction (CR), rapamycin, or acarbose, three of the most extensively characterized lifespan‐extending interventions.^[^ ^42^, ^43^, ^44^ ^]^

**Figure 2:**
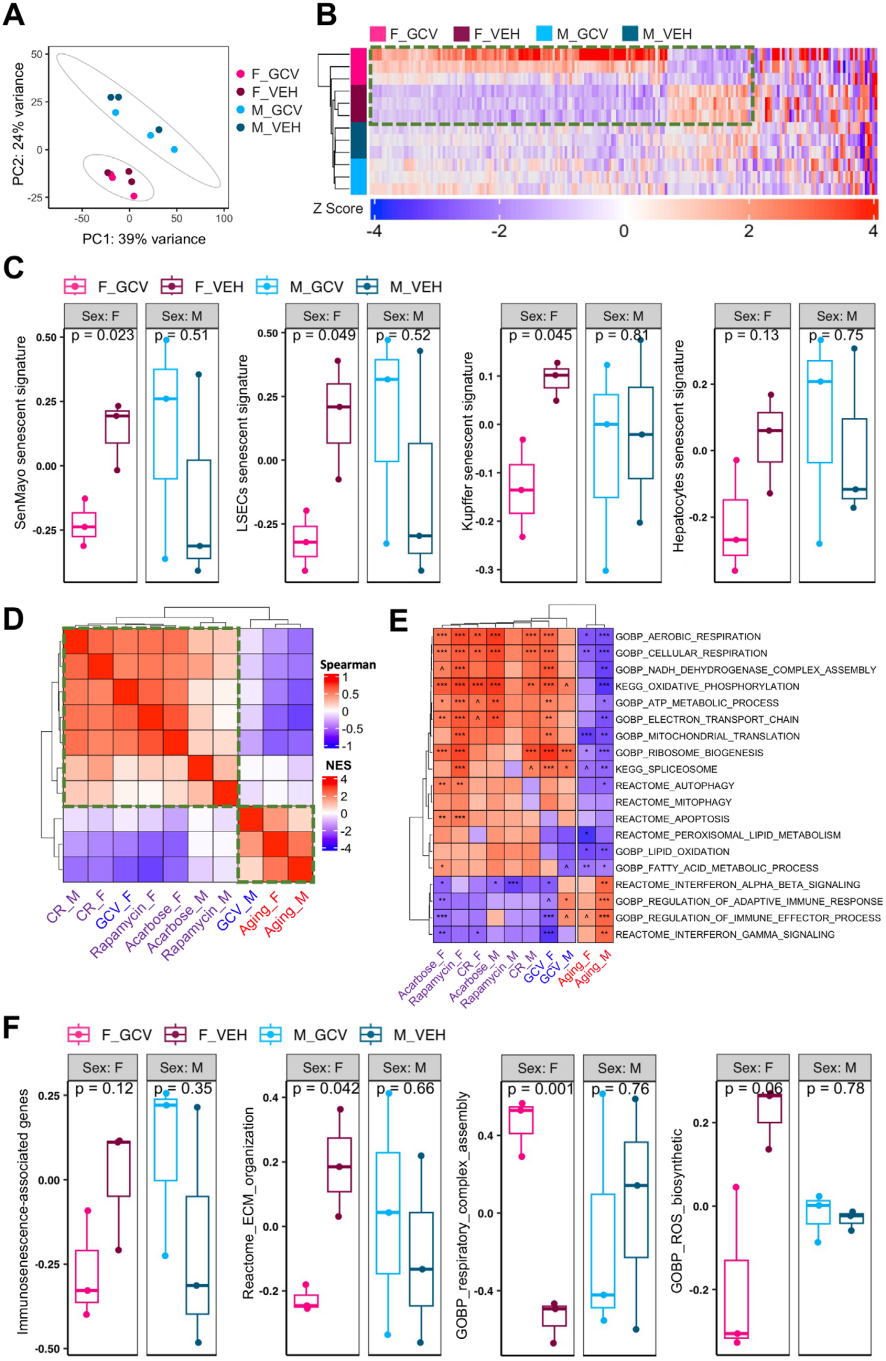
Clearance of p16^+^ cell in aged female livers recapitulates effects of antiaging interventions. A) Principal component analysis (PCA) of bulk RNA‐Seq data from the livers of p16‐3MR female and male mice treated with VEH or GCV (*N* = 3 per group). B) Heatmap of mean‐centered expression values of differentially expressed genes (DEGs) between GCV‐ and VEH‐treated p16‐3MR female and male mice (*N* = 3 per group). A list of DEGs is shown in Table S2 (Supporting Information). C) Gene set variation analysis (GSVA) scores for general (SenMayo) and cell type‐specific (LSECs, Kupffer, Hepatocyte) senescent gene sets in GCV‐ and VEH‐treated p16‐3MR female and male mice. Data were analyzed using a two‐sided Student's *t*‐test (*N* = 3 mice per group). D) Heatmap showing pairwise Spearman correlations based on normalized enrichment scores (NES) from gene set enrichment analysis (GSEA) of the aging process, classical antiaging interventions, and GCV treatment (both sexes). Only gene sets with adjusted *p*‐values < 0.1 were included in the correlation analysis. GSEA was performed on genes ranked by expression changes, and enrichment scores were normalized across gene sets. Gene sets were derived from REACTOME, KEGG, and Gene Ontology (GO Biological Processes) collections from the MSigDB database. Color intensity indicates the strength of correlation between groups. The green box highlights clusters in which mouse groups show stronger correlations within the cluster than with mouse groups outside the cluster. E) Heatmap displaying NES of selected gene sets from GSEA comparing the aging process, antiaging interventions, and GCV treatment (both sexes). The color scale represents the NES values. Significance thresholds: ***p < 0.001, **p < 0.01, *p < 0.05, ^*p* < 0.1 (multiple testing adjusted by Benjamini–Hochberg). The list of enriched functions is presented in Table S4 (Supporting Information). F) GSVA signature changes in key pathways in p16‐3MR female and male mice treated with GCV and VEH (*N* = 3 per group). Data were analyzed using two‐sided Student's *t*‐test.

Aged p16‐3MR mice exhibited a strong transcriptional response to GCV‐induced p16^+^ cell clearance, particularly in females. Indeed, differential expression analysis identified 199 DEGs in GCV‐treated versus VEH‐treated female livers, compared to only 9 DEGs in males (**Figure** **2**A,B; Table S2, Supporting Information). Hierarchical clustering of these DEGs revealed distinct sex‐ and treatment‐specific expression patterns, with GCV‐ and VEH‐treated female samples forming separate clusters from the male samples (Figure 2B). To control for potential off‐target effects of GCV, wild‐type (WT) mice were treated with three cycles of VEH or GCV, followed by liver RNA‐seq. Principal component analysis (PCA) demonstrated clustering primarily by sex, with no clear separation between the treatment groups (Figure S4A, Supporting Information). Correspondingly, differential expression analysis identified only 12 and 7 DEGs in females and males, respectively (adjusted *p* < 0.05; Figure S4B,C, Supporting Information), indicating an insignificant transcriptional impact of GCV in the absence of the p16‐3MR transgene. Collectively, these results confirm that the observed transcriptomic changes in p16‐3MR mice are driven by the targeted elimination of p16⁺ cells rather than the nonspecific effects of GCV.

To validate the effective clearance of senescent cells following GCV administration in p16‐3MR mice, we analyzed RNA‐seq data using established senescence‐associated gene signatures. These include the SenMayo composite signature as well as liver cell type‐specific senescence signatures for liver sinusoidal endothelial cells (LSECs), Kupffer cells, and hepatocytes.^[^ ^39^, ^45^ ^]^ In aged female mice, all four signatures exhibited reduced expression following GCV treatment, with three showing a statistically significant downregulation (Figure 2C). In contrast, no reduction in signature expression was observed in the GCV‐treated males. These results are consistent with previous experimental evidence (Figure 1E) and support more efficient depletion of p16^+^ cells in female p16‐3MR mice in response to GCV.

To evaluate the molecular parallels between p16⁺ cell clearance and established antiaging interventions, we compared liver RNA‐seq profiles from GCV‐treated p16‐3MR mice with publicly available transcriptomic datasets from aged versus young mice and from aged mice treated with interventions such as calorie restriction, rapamycin, or acarbose. For each dataset, we independently performed differential gene expression and gene set enrichment analyses (GSEA), followed by cross‐dataset comparisons (see the Experimental Section). At the gene level, Spearman's correlation analysis revealed strong positive correlations between transcriptional changes in GCV‐treated aged females and those observed in longevity interventions, particularly calorie restriction (both sexes), rapamycin, and acarbose (females) (Figure S5A and Table S3, Supporting Information). Conversely, the gene expression profiles of GCV‐treated females were negatively correlated with aging‐associated transcriptomic signatures. Hierarchical clustering further supported these results, with GCV‐treated female samples clustering closely with antiaging interventions, whereas GCV‐treated male samples clustered with aging profiles (Figure S5A,B, Supporting Information). At the gene set level, these trends were amplified; GCV‐treated females showed significant enrichment of mitochondrial and oxidative phosphorylation‐related pathways, similar to longevity interventions and opposite to aging (Figure 2D,E; Table S4, Supporting Information). Furthermore, immune‐related gene sets were downregulated in both longevity interventions and GCV‐treated females but not in GCV‐treated males.

To further investigate pathway‐level changes associated with p16⁺ cell clearance, we performed gene set variation analysis (GSVA)^[^ ^46^ ^]^ on the liver transcriptomes of GCV‐ and VEH‐treated p16‐3MR mice (Figure 2F). Given the well‐established role of extracellular matrix (ECM) remodeling and fibrosis in hepatic aging,^[^ ^47^, ^48^ ^]^ we assessed ECM‐related gene sets and observed reduced expression in GCV‐treated females. Furthermore, aging‐associated declines in mitochondrial function, characterized by decreased oxidative phosphorylation and increased reactive oxygen species (ROS) production,^[^ ^49^ ^]^ are counteracted by p16^+^ cell clearance. Specifically, GCV‐treated females exhibited transcriptional signatures indicative of enhanced mitochondrial complex assembly and suppressed ROS biosynthesis (Figure 2E,F), suggesting a potential beneficial effect on mitochondrial activity.

These findings demonstrate that p16^+^ cell clearance induces sex‐dependent transcriptional remodeling, with GCV‐treated females exhibiting a molecular profile consistent with enhanced mitochondrial function and reduced ECM‐ and immune cell‐related gene sets.

### 2.3 Proteomic‐Transcriptomic Integration Confirms Female‐Specific Benefits of Eliminating p16^+^ Cells

**Figure 3:**
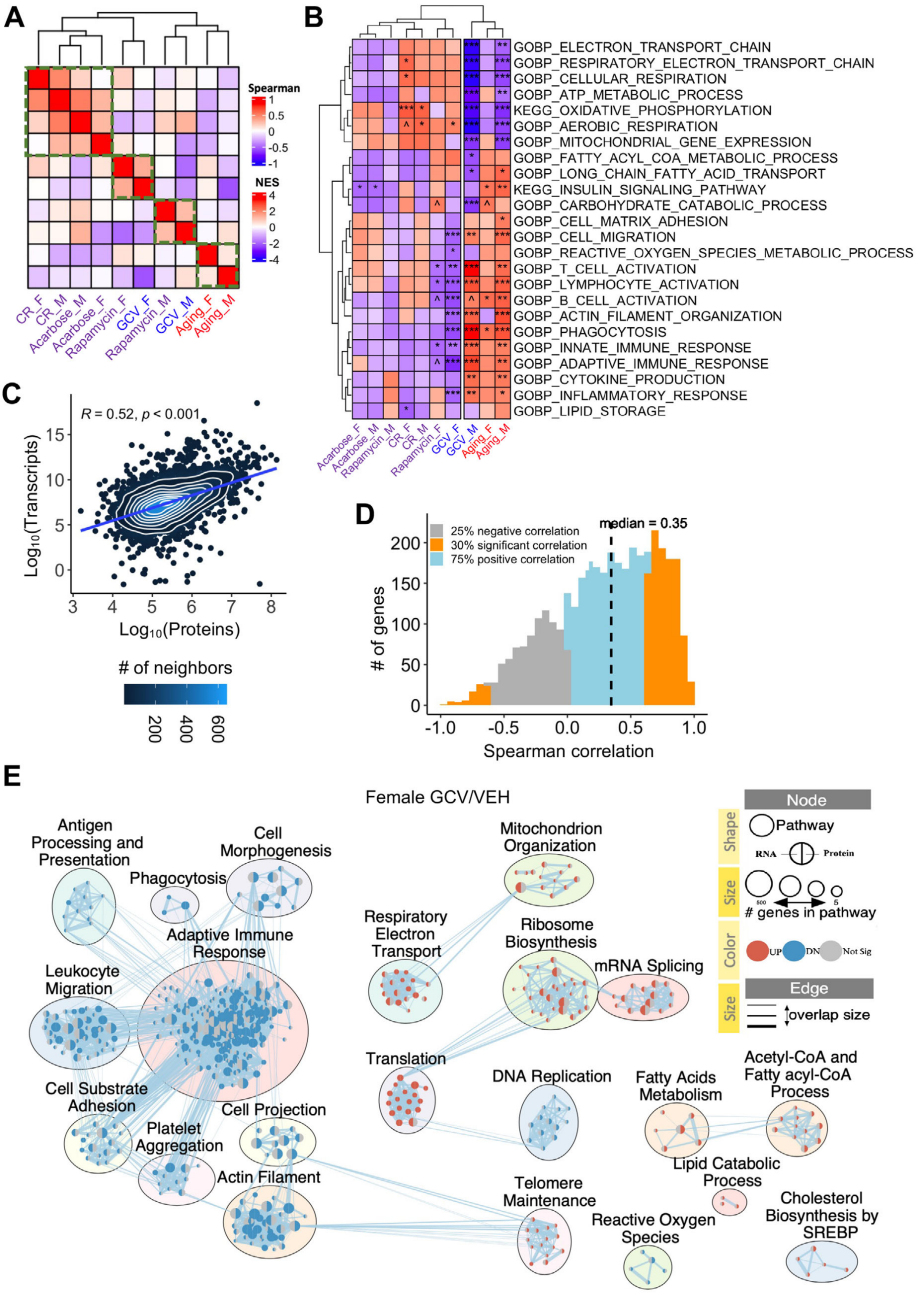
Proteomic analysis highlights a female‐specific benefit of p16^+^ cell clearance. A) Heatmap showing pairwise Spearman correlations of proteomics‐based normalized enrichment scores (NES) from gene set enrichment analysis (GSEA) of the aging process, classical antiaging interventions, and GCV treatment (both sexes). Only gene sets with adjusted *p*‐values < 0.1 were included in the correlation analysis. GSEA was performed on genes ranked by expression changes, and enrichment scores were normalized across gene sets. Gene sets were derived from REACTOME, KEGG, and Gene Ontology (GO Biological Processes) collections from the MSigDB database. Color intensity indicates the strength of correlation between groups. The green box highlights clusters in which mouse groups show stronger correlations within the cluster than with mouse groups outside the cluster. B) Heatmap displaying NES of selected gene sets from proteomics‐based GSEA comparing the aging process, antiaging interventions, and GCV treatment (both sexes). The color scale represents the NES values. Significance levels: ***p < 0.001, **p < 0.01, *p < 0.05, ^*p* < 0.1 (multiple testing adjusted by Benjamini–Hochberg). The list of enriched functions is presented in Table S6 (Supporting Information). C) Density scatter plot showing the log10‐transformed abundance of transcripts and encoded proteins (average across all samples), with linear regression fit (blue line) and Spearman correlation (*R* = 0.52, *p* < 0.001). The samples averaged here are bulk RNA‐Seq datasets from the livers of GCV‐ or VEH‐treated female and male p16‐3MR. Sample‐specific correlations are shown in Figure S4B (Supporting Information). Points colored by local density (number of neighbors). D) Histogram showing the distribution of gene‐specific mRNA‐protein Spearman correlations based on the transcriptomic and proteomic data from livers of GCV‐ or VEH‐treated female and male p16‐3MR mice. The dashed line indicates the median correlation coefficient. E) Functional network showing GSEA results of both transcriptomic and proteomic comparisons of GCV versus VEH treatment in p16‐3MR female mice. Nodes represent the gene set (size ∝ set size) colored in the enrichment direction (red, positive; blue, negative; gray, not significant; left, transcriptome; right, proteome). Edges indicate gene overlap between sets. Gene sets with related functions were grouped and labeled for clarity.

To assess protein‐level changes induced by p16⁺ cell clearance, we conducted untargeted proteomic profiling of livers from male and female mice treated with either VEH or GCV. Differential protein expression analysis (Table S5, Supporting Information) revealed reductions in several senescence‐associated proteins including β‐galactosidase (GLB1), regulators of the SASP such as the NF‐κB (MSK1, RELA, NF‐κB2, NF‐κB1) and mTOR (RHEB, MTOR, RPTOR), as well as SASP effectors (ICAM1, PECAM1). In addition, we observed altered protein expression levels for several rejuvenation‐related genes, including inflammation reduction, mTOR signaling, protein folding, mitochondrial mass and pro‐oxidant genes (Figure S6A, Supporting Information). Similar to our transcriptomic analysis, we performed GSEA and calculated Spearman correlations using normalized enrichment scores (NES), comparing our proteomic data to independent datasets from aging and various antiaging interventions^[^ ^20^, ^21^ ^]^ (Table S6, Supporting Information). Notably, these reference datasets were distinct from those used in the RNA‐seq comparisons. GCV‐treated females exhibited the strongest positive correlation with rapamycin‐treated females, whereas GCV‐treated males were most closely aligned with rapamycin‐treated males (**Figure** **3**A). This finding is consistent with the sex‐specific benefits of rapamycin^[^ ^17^ ^]^ and its ability to reduce multiple senescence‐associated biomarkers, including the SASP.^[^ ^50^ ^]^ The gene set enrichment patterns mirrored those observed at the transcriptomic level (Figure 3B). In females, GCV treatment led to positive enrichment of aerobic respiration pathways and negative enrichment of immune‐related pathways, changes opposite to those associated with aging. However, in males, GCV did not have the same effect on these pathways, underscoring a sex‐specific divergence in molecular response.

After benchmarking our data against public datasets, we evaluated the concordance between transcriptomic and proteomic profiles within our own cohort by analyzing both gene‐ and gene set‐level relationships. Previous studies have reported an age‐associated decline in mRNA–protein coupling in humans.^[^ ^51^, ^52^ ^]^ However, within our experimental datasets, transcript and protein expression levels were globally correlated (*R* = 0.52, *p* < 0.001; Figure 3C), indicating moderate but statistically significant concordance. At the single‐gene level, 75% of genes with matched transcript and protein data exhibited positive Spearman correlations, with 30% reaching statistical significance and a median correlation coefficient of 0.35 (Figure 3D). These includes the differentially expressed proteins identified in senescence‐associated pathways (Figure S6B, Supporting Information).

At the gene set level, we integrated GSEA results from both transcriptomic and proteomic datasets in female mice treated with GCV versus vehicle. This analysis revealed a combination of shared and assay‐specific pathway enrichments (Figure 3E). Pathways commonly downregulated across both omics layers include adaptive immune response, antigen processing and presentation, phagocytosis, cell–substrate adhesion, and actin filament organization, contrasting aging‐associated increases in hepatic immune cell infiltration.^[^ ^38^, ^53^ ^]^ In contrast, pathways related to respiratory electron transport and translation are consistently upregulated following p16⁺ cell clearance, opposing the age‐associated decline in ribosome occupancy and protein synthesis capacity.^[^ ^54^ ^]^ Transcriptome‐specific enrichment included mitochondrial organization, ribosome biogenesis, and mRNA splicing, while proteome‐specific enrichment highlighted DNA replication, telomere maintenance, reactive oxygen species metabolism, and lipid metabolism.

Overall, the proteomic analysis confirmed the transcriptomic trends, strengthening the evidence that eliminating p16⁺ cells promote gene expression patterns consistent with enhanced mitochondrial function and lower inflammatory activity, particularly in females.

### 2.4 Convergent Transcriptomic Signatures Across Distinct Senescent Cell Clearance Strategies

Despite the growing interest in senescence‐targeting therapies, a unified transcriptional signature following senescent cell clearance has not yet been established. To address this gap, we integrated publicly available liver RNA‐seq datasets from murine models treated using distinct senolytic or senomorphic strategies. Specifically, we analyzed the data from ouabain‐treated mice^[^ ^53^ ^]^ and miR‐302b‐treated mice.^[^ ^55^ ^]^ Ouabain induces apoptosis in diverse senescent cell types, including oncogenes, therapies, and replicative stress‐induced senescent cells, both in vitro and in vivo.^[^ ^53^, ^56^ ^]^ In contrast, miR‐302b reactivates the cell cycle in replicative senescent cells by repressing *Cdkn1a* and *Ccng2*.^[^ ^55^ ^]^

**Figure 4:**
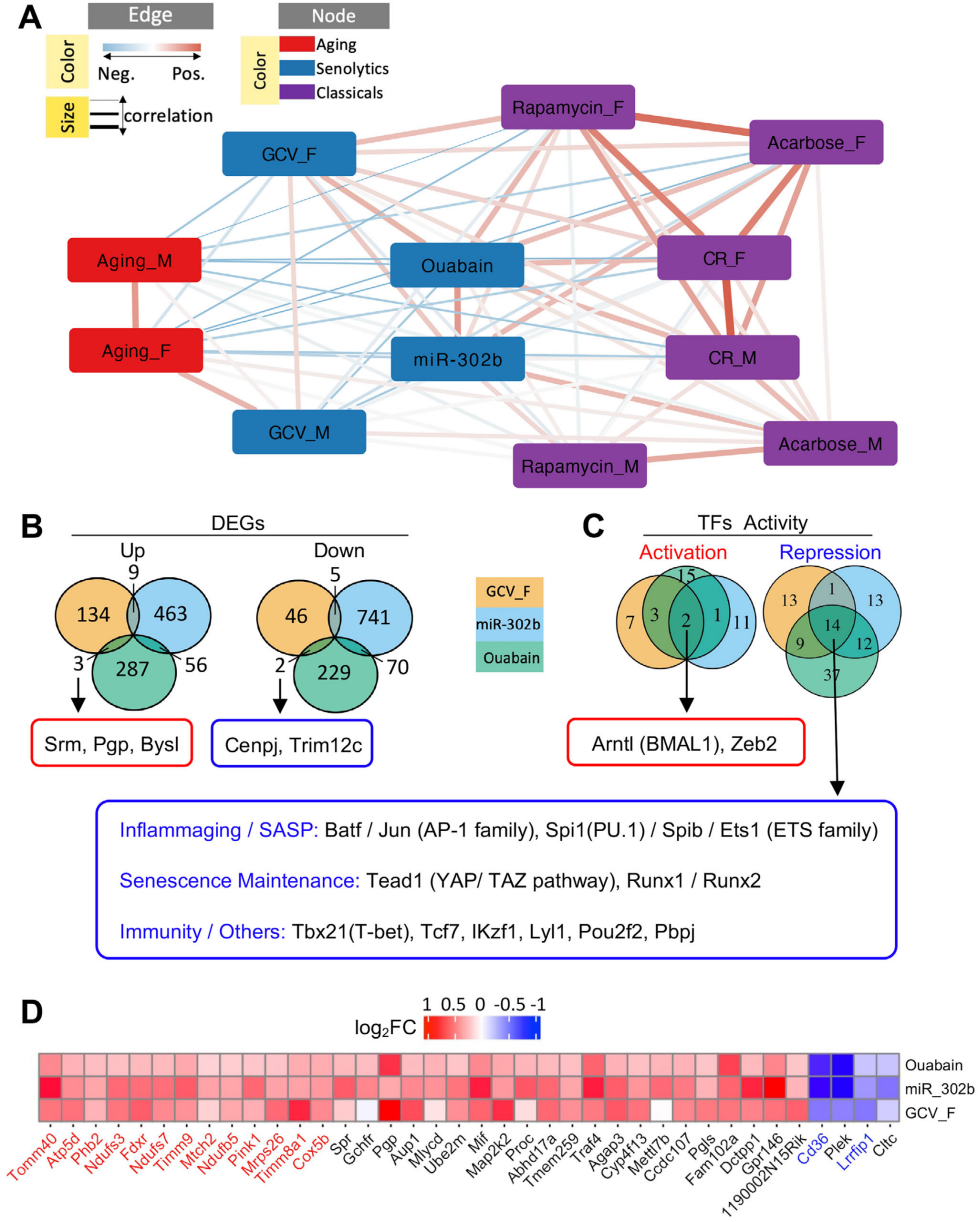
Common transcriptional signatures of diverse senescent cell clearance approaches. A) Network showing Spearman's correlation based on gene expression changes under diverse experimental conditions. Only genes that were significantly changed in at least one condition were included in the correlation calculation (p.adj < 0.01). Nodes represent experimental conditions colored by category (red: aging process; purple: classical antiaging interventions; blue: senescent cell clearance, including both sexes). Edges indicate correlations between gene expression profiles (edge width ∝ absolute value of correlation coefficients; red: positive; blue: negative). B) Venn diagram showing the overlap of differentially expressed genes (DEGs) (p.adj < 0.05) in diverse senescent cell clearance approaches: GCV treatment in p16‐3MR females (*N* = 3 mice/group, ≈28 months, self‐generated), miR‐302b in C57BL/6 mice (*N* = 3 mice/group, original data from Bi et al.^[^ ^55^ ^]^), and ouabain treatment in C57BL/6 female mice (*N* = 6 mice/group, 24 months, original data from Guerrero et al.^[^ ^53^ ^]^). All samples were obtained from the liver tissue. C) Venn diagram showing common transcription factors (TFs) across all three clearance approaches grouped by activation or repression activities. A list of transcription factors is presented in Table S7 (Supporting Information). D) Heatmap showing log_2_ fold‐changes for genes consistently altered across all senescence clearance methods based on random‐effects meta‐analysis (*p* < 0.01) and leave‐one‐out sensitivity analysis (max *p* < 0.1). Mitochondrial genes (red text) and inflammatory genes (blue text) have been highlighted. The complete list of genes is presented in Table S8 (Supporting Information).

We first assessed the gene‐level similarities across interventions by calculating the Spearman correlations of the differential expression profiles. As expected, aging correlated negatively with the interventions, except in GCV‐treated males. Among the senolytic treatments, GCV‐treated females showed the strongest correlation with ouabain‐treated mice, and ouabain and miR‐302b treatments exhibited high concordance (**Figure** **4**A).

Next, we examined the overlap of differentially expressed genes (adjusted *p* < 0.05) between treatments (Figure 4B). While no single gene was commonly regulated across all three interventions, GCV‐ and Ouabain‐treated females shared six genes, including three upregulated (*Srm*, *Pgp*, *Bysl*) and two downregulated (*Cenpj*, *Trim12c*). Notably, *Srm*, which encodes spermidine synthase, has been linked to longevity and the attenuation of cellular senescence.^[^ ^57^, ^58^ ^]^ GCV‐ and miR‐302b‐treated mice shared 15 regulated genes, including *Entpd1*, a marker of T cell exhaustion, *Nmrk1*, a key enzyme in NAD⁺ biosynthesis, and *Mtres1*, which maintains mitochondrial transcript stability. These shared gene‐level changes suggest the possibility that certain pathways might be key regulator of senolytic efficacy.

As strict intersection‐based gene overlap may overlook subtle but consistent expression changes, we applied complementary strategies to capture convergent transcriptomic remodeling: transcription factor (TF) activity inference and meta‐analysis. Using the DoRothEA TF‐target database (v1.8.0)^[^ ^59^ ^]^ and DecoupleR framework (v2.9.7),^[^ ^60^ ^]^ we inferred TF activity from gene expression profiles across the three senescence clearance datasets. We identified two TFs with shared activation, *Arntl* and *Zeb2*, and 14 TFs with repressed activities (Figure 4C; Table S7, Supporting Information).

*Arntl* encodes BMAL1, a master circadian regulator that plays critical roles in longevity, senescence suppression, and mitochondrial function. Its expression declines with age,^[^ ^61^ ^]^ and BMAL1 deficiency shortens lifespan and accelerates aging.^[^ ^62^ ^]^ Mechanistically, BMAL1 stabilizes heterochromatin, promotes DNA repair, and maintains mitochondrial health.^[^ ^63^, ^64^, ^65^ ^]^ *Zeb2*, although less studied in the context of aging, has emerging links to immune and developmental regulation.

Repressed TFs fell into three functional categories: (1) regulators of inflammaging and the SASP (e.g., *Jun*, *Batf*, *Spi1*, *Spib*, *Ets1*), (2) TFs involved in senescence maintenance (e.g., *Tead1*, *Runx1*, *Runx2*), and (3) TFs related to immune cell identity and function (e.g., *Tbx21*, *Tcf7*, *Ikzf1*, *Lyl1*, *Pou2f2*, *Pbpj*). Many of these factors are implicated in promoting or sustaining senescence. For example, *Tead1* supports the survival of senescent cells through YAP‐TEAD signaling,^[^ ^66^ ^]^ and *Ets1* promotes *p16^Ink4a^* expression.^[^ ^67^, ^68^ ^]^ Repression of these TFs suggests the potential for partial suppression of senescence‐supportive transcriptional programs following cell clearance.

To identify consistently regulated genes across treatments, we conducted a random‐effects meta‐analysis with leave‐one‐out (LOO) sensitivity testing (see Methods). Using stringent thresholds (meta *p* < 0.01; max LOO *p* < 0.1), we identified 38 genes that were consistently regulated across the studies (Figure 4D; Table S8, Supporting Information). Notably, approximately one‐third of the up‐regulated genes were associated with mitochondrial function. Among the downregulated genes, *Cd36*, a known promoter of SASP and inflammation,^[^ ^69^, ^70^, ^71^ ^]^ emerged as a consistent hit. Another repressed gene, *Lrrfip1*, encodes a cytosolic nucleic acid sensor that activates type I interferon responses,^[^ ^72^ ^]^ and its downregulation may contribute to reduced mitochondrial stress and immune activation post clearance.

Collectively, these findings revealed a core set of transcriptional regulators and pathways that are consistently modulated across different senescent cell clearance strategies, suggesting shared molecular responses rather than a fully conserved rejuvenation program at the tissue level.

## 3 Discussion

In this study, we demonstrate that aged female mice harbor a significantly higher burden of *Cdkn2a⁺*, primarily p16⁺, cells than age‐matched males. This observation was consistently supported by multiple orthogonal approaches, including public single‐nucleus RNA‐seq (snRNA‐seq), bulk RNA‐seq, GLF16 senescence staining, and immunostaining for the SASP factor IL1α. Although most p16⁺ cells exhibit features of senescence, they represent a heterogeneous population, and not all are fully senescent. Nevertheless, the higher prevalence of these cells in aged females suggests that p16‐driven senescence or p16 expression may be more prominent in the female aging trajectory.

The mechanisms underlying this sex difference are likely multifactorial. First, immune surveillance may decline more rapidly in females with age. For example, aging‐associated increases in asparagine‐linked glycans, which are known inhibitors of T cell activity, are more pronounced in females.^[^ ^73^ ^]^ In line with this, snRNA‐seq data revealed accelerated accumulation of immunosenescent populations in female livers, including Gzmk⁺ CD8⁺ T cells, γδ T cells, memory CD4⁺ T cells, Kupffer cells, inflammatory monocytes, and aging‐associated B cells (Figure S7, Supporting Information). Second, intrinsic cellular differences may also contribute, with female cells potentially relying more on *Cdkn2a*/p16‐mediated senescence as a tumor‐suppressive mechanism, whereas male cells may preferentially accumulate DNA damage, leading to apoptosis or transformation.^[^ ^74^, ^75^ ^]^ These findings are consistent with prior work showing sex‐dependent variation in senescent cell burden.^[^ ^37^ ^]^

Using the p16‐3MR mouse model, we selectively ablated p16⁺ cells via ganciclovir (GCV) treatment. RNA‐seq analysis of wild‐type mice confirmed minimal off‐target effects of GCV on hepatic gene expression, indicating that transcriptomic and physiological changes in GCV‐treated p16‐3MR mice reflected bona fide p16⁺ cell clearance. This was further validated by GLF16 staining, RT‐qPCR for p16, IL1α staining, and reduction in senescence‐associated gene signatures.

Importantly, the physiological benefits of p16⁺ cell clearance were largely restricted to aged females, who displayed improved skin regeneration, grip strength, and attenuated liver damage. In contrast, male mice exhibited minimal transcriptional and functional responses, consistent with their lower baseline burden of p16⁺ cells. While these data support a sex‐specific effect, they do not exclude the possibility that males could benefit from alternative or optimized senolytic strategies, such as adjusted dosing, extended treatment, or combinatorial approaches. This may also reflect underlying differences in the dominant senescence pathways between sexes: males may engage the DNA damage–p53–p21 axis more strongly, whereas females rely more on replicative or stress‐induced p16 activation. The influence of sex hormones on these divergent senescence programs remains to be elucidated but represents an intriguing direction for future work.

Integrated transcriptomic and proteomic analyses of female liver tissue revealed an upregulation of genes associated with mitochondrial metabolism and oxidative phosphorylation, alongside suppression of immune and inflammatory pathways. These shifts oppose those observed in natural aging and parallel transcriptional signatures induced by longevity‐promoting interventions. Interestingly, although rapamycin and acarbose are known to show sex‐specific efficacy—favoring females and males, respectively^[^ ^16^, ^17^ ^]^—our data revealed stronger molecular correlations between male GCV‐treated livers and those treated with either drug. This unexpected result could reflect sex‐specific differences in drug metabolism, hepatic physiology, or in the identity and senescent status of p16⁺ cells, warranting further investigation.

The analytical framework used here—integrating multi‐omics data from senolytic interventions with established aging and longevity signatures—can be broadly applied to evaluate novel pharmacological, dietary, or lifestyle interventions.

Our study has several limitations. The absence of a clear response in males may reflect incomplete clearance of p16⁺ cells rather than true biological insensitivity. We also did not detect robust *Cdkn2a* or 3MR transgene expression in RNA‐seq data, likely due to the low abundance of these transcripts and modest sequencing depth (5–10 million reads per sample). Furthermore, while integrating public datasets strengthens generalizability, it introduces potential batch effects. However, our comparative strategy, which shows that GCV‐specific profiles are consistent to trends across aging and antiaging datasets, helps mitigate this. Prior studies suggest that biological variables outweigh dataset origin in shaping transcriptomic outcomes.^[^ ^76^, ^77^ ^]^ Finally, while we identified promising gene and transcription factor candidates, functional validation will be required to confirm their causal roles in aging and senescence regulation.

Beyond our core findings, integrative analysis across independent senescence‐targeting studies revealed a shared set of transcriptional regulators and pathways modulated by senescent cell clearance. These include genes involved in mitochondrial function, SASP regulation, and immune signaling, several of which may represent candidate targets for senotherapeutic development. For instance, *Srm*, a gene linked to spermidine metabolism and healthspan extension, was consistently upregulated, whereas *Cd36* and *Lrrfip1*, both implicated in pro‐inflammatory signaling, were downregulated. Nevertheless, distinct clearance methods likely have different off‐target effects, which should be considered when interpreting shared molecular patterns.

In summary, our findings reveal sex‐specific molecular and functional benefits of p16⁺ cell clearance, particularly in aged females, marked by improved tissue repair, metabolic health, and reduced inflammation. These results advance understanding of p16⁺ cell biology, emphasizing both their heterogeneity and context‐dependent roles, and highlight the importance of sex as a critical biological variable in the design and evaluation of senescence‐targeting therapies.

## 4 Experimental Section

### Single‐Nucleus and Bulk RNA‐Seq Data Analysis

A publicly available single‐nucleus RNA sequencing (snRNA‐seq) dataset from Zhang et al.^[^ ^38^ ^]^ was analyzed to quantify the temporal dynamics of *Cdkn2a^+^* cell proportions in wild‐type female and male mice across five age groups (3, 6, 12, 16, and 23 months). As the dataset had already undergone quality control (removal of low‐quality cells and doublets) by the original authors, no further filtering was applied.

The analysis focused on eight core tissues: the liver, kidney, heart, stomach, lung, brown adipose tissue, inguinal white adipose tissue, and gonadal white adipose tissue. Cells with nonzero *Cdkn2a* expression were classified as *Cdkn2a^+^* cells. To evaluate the overall age‐related trends, data from all tissues were aggregated at different time points. Tissue‐ and sex‐specific *Cdkn2a^+^* cell proportions at 23 months of age were also examined. For age‐ and sex‐specific analyses of the liver tissue, differences in *Cdkn2a^+^* cell proportions between the sexes were assessed using Pearson's chi‐squared test with Yates’ continuity correction.

To validate sex‐related differences in senescence signatures, the Tabula Muris Senis bulk RNA‐seq liver dataset was analyzed. GSVA was performed using the SenMayo senescence gene set^[^ ^39^ ^]^ and the GSVA algorithm.^[^ ^46^ ^]^ All visualizations were generated using the ggplot2 package in R (v4.3.1).

### Mouse Models, Treatments, and Experimental Design

All animal procedures were conducted at the Central Animal Facility (CDP) of the University Medical Center Groningen (UMCG) under standard housing conditions. All experiments were approved by the Central Authority for Scientific Procedures on Animals (CCD, The Netherlands; License numbers AVD105002015339, AVD1050020184807, and AVD10500202115445). The *p16‐3MR* transgenic mouse model^[^ ^25^ ^]^ was used to investigate the effects of *p16* **^+^** cell clearance. Both male and female mice were allowed to age naturally. Starting at 16–18 months of age, mice received monthly treatments with either vehicle (VEH; pH11 water) or ganciclovir (GCV; 25 mg kg^−1^ in pH11 water) administered intraperitoneally for five consecutive days. Assays were performed at defined time points during the course of treatment (Figure S1A, Supporting Information).

At ≈21 months of age, a subset of the treated mice (*N* = 3–5 per group) was sacrificed. Liver tissue was collected and sectioned for GLF16 staining,^[^ ^40^ ^]^ plasma was obtained for alanine aminotransferase (ALT) assays, and the dorsal skin was processed for histological analysis (Dutch Molecular Pathology Center, Utrecht, The Netherlands). At ≈24 months of age, the mice were tested for grip strength using a digital grip strength meter (Columbus Instruments), and skin regenerative capacity was evaluated using a standardized 4 mm punch wound healing assay (*N* = 5–6 per group). At ≈28 months of age, a final subset of mice was sacrificed, livers, kidneys and lungs were harvested for RT‐qPCR and livers were also used for both bulk RNA sequencing and proteomic analysis. As an additional control, a cohort of age‐matched wild‐type (WT) male and female mice began receiving monthly VEH or GCV treatment from 6 months of age. After 3 months, livers were collected for RNA sequencing analysis (*N* = 3 per group).

### RNA Isolation, Library Preparation, and Sequencing

Total RNA was extracted from mouse liver tissue using the ISOLATE II RNA Mini Kit (Bioline, cat# BIO‐52073) following the manufacturer's protocol. RNA concentration was measured using a NanoDrop spectrophotometer (Thermo Fisher Scientific) and RNA integrity was assessed using the Agilent TapeStation system. RNA‐seq libraries were generated at the ERIBA Research Sequencing Facility (UMCG, Groningen, The Netherlands) using the Smart‐3SEQ protocol^[^ ^78^ ^]^ with 15 ng of input total RNA per sample. Libraries were pooled and sequenced on the Illumina NovaSeq X Plus PE150 platform (Novogene, Munich, Germany).

### RNA‐Seq Data Processing and Differential Expression Analysis

Raw sequencing reads were quality checked using FastQC (v0.11.9)^[^ ^79^ ^]^ and trimmed for adapters using TrimGalore (v0.6.10) with default parameters. Clean reads were pseudoaligned to a pre‐built mouse decoy transcriptome index (via refgenie) using Salmon (v1.10.3)^[^ ^80^ ^]^ with the flags gcBias, seqBias, and validateMappings. Transcript‐level quantification was aggregated into gene‐level counts using tximport (v1.24.0).^[^ ^81^ ^]^ Genes with <3 reads were excluded. Principal component analysis was performed using the prcomp function in R. Differential gene expression was conducted using DESeq2 (v1.36.0),^[^ ^82^ ^]^ applying the Benjamini–Hochberg (BH) correction for multiple testing. Log_2_ fold‐change shrinkage was applied using ashr,^[^ ^83^ ^]^ and genes with adjusted *p* < 0.05 were considered statistically significant. To evaluate senescence‐targeting effects of GCV treatment, gene set variation analysis (GSVA)^[^ ^46^ ^]^ was performed using two curated senescence signatures: the SenMayo gene set^[^ ^39^ ^]^ and a liver‐specific senescence signature.^[^ ^45^ ^]^

### Comparative Transcriptomic Analyses of Antiaging Interventions

To contextualize the transcriptional impact of *p16* ^+^ cell clearance, publicly available liver RNA‐seq datasets were analyzed: natural aging^[^ ^18^ ^]^ (GSE132040) and three canonical antiaging interventions—calorie restriction, rapamycin, and acarbose^[^ ^19^ ^]^ (GSE131901). Raw count matrices were downloaded from the NCBI Gene Expression Omnibus. Differential expression analysis of each intervention was performed using the pipeline described above. At the gene level, expression changes were computed as follows:

(1) $$-\mathrm{lo}g_{10}\left(\mathrm{adjusted}\;\;p\;\mathrm{value}\right)\times \mathrm{sgn}\left(\mathrm{lo}g_{2}\;\mathrm{Foldchange}\right)$$

Only the genes shared across all studies (*n* = 16076) were retained. Genes with absolute transformed scores >2 under at least one condition (*n* = 4104) were used for downstream analyses. Hierarchical clustering based on Spearman's correlation coefficients among the experimental conditions was conducted and visualized as a heatmap. At the pathway level, GSEA^[^ ^84^ ^]^ was performed using the clusterProfiler package (v4.2.1)^[^ ^85^ ^]^ in pre‐ranked mode. Gene sets were derived from REACTOME, KEGG, and Gene Ontology (GO Biological Processes) collections from the MSigDB databases. BH‐corrected *p*‐values were calculated separately for each database. Gene sets with adjusted *p* < 0.1 in any condition were included for further correlation and clustering analysis. Representative enriched pathways were manually selected and visualized based on NES using heatmaps.

### Proteomics Sample Preparation and Mass Spectrometry

A total of 25 µg of protein from mouse liver lysates was mixed with LDS loading buffer (final volume 40 µL) and briefly electrophoresed (5 min at 100 V) on precast 4%–12% Bis‐Tris gels (Thermo Fisher Scientific). The gels were stained with BioSafe Coomassie G‐250 (Bio‐Rad), and protein bands encompassing the entire molecular weight range were excised. Excised gel bands were cut into ≈1 mm^3^ fragments and sequentially washed with 30% and 50% (v/v) acetonitrile in 100 × 10^−3^ m ammonium bicarbonate (in milliQ‐water) for 30 min at room temperature with shaking (500 rpm), followed by a final wash with 100% acetonitrile (5 min). Gel pieces were dried at 37 °C. Proteins were reduced with 30 µL of 10 × 10^−3^ m dithiothreitol in 100 × 10^−3^ m ammonium bicarbonate (30 min at 55 °C) and alkylated with 30 µL of 55 × 10^−3^ m iodoacetamide in 100 × 10^−3^ m ammonium bicarbonate (30 min in the dark, RT). After an additional wash with 100% acetonitrile (30 min, 500 rpm), the gel pieces were dried and incubated overnight at 37 °C with 30 µL of 8.3 ng µL^−1^ sequencing‐grade modified trypsin (V5111; Promega). The peptides were extracted using 50 µL of 75% (v/v) acetonitrile and 5% (v/v) formic acid (20 min at RT, 500 rpm), dried under vacuum, and reconstituted in 500 µL of 0.1% (v/v) formic acid.

### LC‐MS/MS Acquisition and Data Processing

Peptide mixtures (20 µL per sample) were analyzed on a quadrupole‐Orbitrap mass spectrometer (Orbitrap Exploris 480, Thermo Scientific)^[^ ^86^ ^]^ equipped with a nano‐electrospray source and coupled to an Evosep One LC system (Evosep). Peptides were separated on a 15 cm × 150 µm, 1.5 µm particle size column (EV1137, Evosep) using the 30 samples‐per‐day (30SPD) method. The mobile phases were buffer A (0.1% formic acid in Milli‐Q water) and buffer B (0.1% formic acid in acetonitrile). The mass spectrometer was operated in positive ion mode with data‐independent acquisition (DIA) using 16 m/z isolation windows across a precursor range of 400–1000 m/z. Scans alternated between compensation voltages of −45 and −60 V, with three scheduled MS1 scans per cycle. Raw data were processed using Spectronaut (v16.0.220606, Biognosys) in the direct DIA workflow against the Mouse SwissProt database (17021 entries). Default settings were used, except for MS1‐based quantification. Q‐value filtering was set to “classic,” normalization was performed locally, and the missing values were not imputed.

### Comparative Proteomics Analysis and Functional Interpretation

To assess the anti‐senescent and antiaging effects of GCV treatment, the proteomic profiles of treated male and female mouse livers were compared with publicly available datasets from the natural aging process^[^ ^21^ ^]^ and canonical antiaging interventions.^[^ ^20^ ^]^ Public data, including fold changes and adjusted *p*‐values for liver proteomes, were retrieved from Figshare (https://doi.org/10.6084/m9.figshare.19765849) and the associated supplementary files.

Functional enrichment and clustering analyses were performed as previously described for transcriptomic data. Briefly, proteins with significant changes in abundance were analyzed for pathway enrichment using GSEA, and hierarchical clustering was performed using Spearman correlations to evaluate condition‐level similarities across interventions and aging.

### Multi‐Omics Integration and Correlation Analysis

This study assessed the relationship between transcriptomic and proteomic data across multiple dimensions: gene‐, pathway‐, and individual mouse‐level comparisons. For gene‐level integration, transcript and protein abundance values were log‐transformed and visualized using scatter density plots. Spearman correlation coefficients were calculated for genes with matched expression data in both datasets and were visualized as a histogram to assess overall concordance.

At the pathway level, enrichment results from RNA‐seq and proteomics analyses were integrated using Enrichment Map^[^ ^87^ ^]^ in Cytoscape (v3.9.1).^[^ ^88^ ^]^ Pathways sharing overlapping genes were connected and related pathways were clustered and annotated using AutoAnnotate.^[^ ^89^ ^]^ The cluster arrangements and labels were manually curated to enhance clarity and minimize redundancy.

To evaluate mRNA–protein expression concordance at the sample level, we calculated Pearson correlation coefficients between the transcriptomic and proteomic profiles for each of the 11 mice with matched data. This analysis enabled the assessment of sample‐wise coupling or decoupling between the transcript and protein expression patterns.

### Meta‐Analysis and Transcription Factor Activity Inference

RNA‐seq datasets from three independent studies, miR‐302b,^[^ ^55^ ^]^ Ouabain,^[^ ^53^ ^]^ and this study, were individually analyzed using DESeq2 (v1.36.0)^[^ ^82^ ^]^ for differential gene expression. Gene‐level associations among aging, GCV treatment, two senolytic compounds, and three classical antiaging interventions were calculated as previously described and visualized using Cytoscape.^[^ ^88^ ^]^

As no genes were significantly differentially expressed across all three datasets based on adjusted *p*‐values, two complementary strategies were employed to identify shared gene signatures and regulatory activities. First, a meta‐analysis of log_2_ fold change (log_2_FC) values and corresponding standard errors (SEs) was performed. Second, a transcription factor (TF) activity analysis across studies was conducted.

For meta‐analysis, a random‐effects model was applied using the metafor R package (v4.8‐0).^[^ ^90^ ^]^ Gene‐level log_2_FCs and SEs obtained from DESeq2 were modeled using the rma function with the Paule–Mandel estimator. Heterogeneity across studies was quantified using the τ^2^ and *I* ^2^ statistics. Genes with a meta‐analysis *p*‐value < 0.01 were considered statistically significant (*n* = 167). To evaluate the robustness of these findings, a leave‐one‐out (LOO) sensitivity analysis was performed, iteratively excluding one study at a time. Genes with a maximum *p*‐value < 0.1 across all three LOO models were defined as robust. The final robust gene signature (*n* = 38) met both criteria: (1) meta‐analysis, *p* < 0.01, and (2) robustness in LOO analysis. These genes were visualized using the pheatmap R package (v1.0.12).

To infer transcription factor activity, decoupleR (v2.9.7)^[^ ^60^ ^]^ along with DoRothEA (v1.8.0) was used,^[^ ^59^ ^]^ focusing on confidence levels A, B, and C. TF activity was estimated for each dataset and filtered based on a significance threshold of *p* < 0.05. Sixteen transcription factors with consistently significant activity across all three studies were identified. Overlaps were visualized using the VennDiagram (v1.7.3).^[^ ^91^ ^]^

### RT‐qPCR

For total RNA isolation from the liver, lung, and kidney tissues, the Isolate II RNA Mini Kit (Cat#BIO‐52073, Bioline) was used. For reverse transcription, around 1000 ng RNA was transcribed into cDNA using the kit (Cat# 4368813, Applied Biosystems). Real‐time qPCR was performed using the GoTaq^®^ qPCR Master Mix (Cat# A6002, Promega). *Tuba1a* was used as housekeeping reference to normalize the relative expression of the target genes. Primers used are listed below:

**Table:** 

*Tuba1a*	Forward	ctggaacccacggtcatc
	Reverse	gtggccacgagcatagttatt
*p16*	Forward	aatctccgcgaggaaagc
	Reverse	gtctgcagcggactccat

### Immunofluorescence in Tissues

The collected tissues were fixed at 4 °C with 4% paraformaldehyde (PFA, Thermo Fisher Scientific #28908) overnight and then embedded into paraffin blocks and sliced into 4 µm slides. On the day of staining, paraffin embedded tissue sections were incubated at 60 °C for 1 h and deparaffinized by washing with xylene (15 min for twice), 100% ethanol (5 min), 96% ethanol (5 min), 70% ethanol (5 min), and water (3 min for three times). For antigen retrieval, slides were boiled for 15 min in citrate buffer (pH 6), cooled down to room temperature and then washed with PBS. Immunofluorescence staining was conducted using standard protocols. Briefly, slides were blocked with 1% BSA at 4 °C overnight, after three times of washes, slides were permeabilized by 0.3% Triton‐X 100 for 4 min and then incubated with primary antibody (IL1α, rabbit, Invitrogen PA5‐89037) for 1 h at room temperature. Following 1 h secondary antibody (Alexa Fluor 647, A21245) incubation at room temperature, tissue autofluorescence was diminished using TrueVIEW autofluorescence quenching kit (Vector, SP‐8400‐15). Slides were then counterstained with DAPI for 5 min and mounted with antifade mounting media (Invitrogen, P36934). Images were acquired using Leica Thunder microscope.

### Statistical Analysis

Statistical analyses were carried out using GraphPad Prism v10 (for functional data) and R v4.2.1 (for omics data). Continuous variables are presented as dot plots with mean ± standard deviation or median ± interquartile range, as indicated in the figure legends, and compared using Student's *t*‐test or two‐way ANOVA as appropriate. Discrete variables are reported as proportions with total counts, and differences between groups were assessed using two‐sided Pearson's chi‐squared test. A *p*‐value < 0.05 was considered statistically significant. Further details are provided in the figure legends.

## Conflict of Interest

M.D. is a founder and shareholder of Cleara Biotech and advisor for Oisin Biotechnologies and Rubedo Life Sciences. The M.D. laboratory received funding from Ono Pharmaceuticals. None of the companies mentioned above were involved in this study. The other authors have no conflicts of interest to declare.

## Author Contributions

Y.L. and B.W. contributed equally to this work. Y.L., B.W., and M.D. conceptualized the study. Y.L., B.W., and M.D. devised the methodology. Y.L., B.W., M.H., and J.C.W. carried out the investigation. M.D. supervised the project. Y.L., B.W., and M.D. wrote the original paper draft. M.D. acquired the funding and managed the project. Y.L., B.W., and M.D. reviewed and edited the manuscript.

## Supporting information



Supporting Information

Supporting Information

## Data Availability

The bulk RNA‐seq dataset generated in this study was deposited in the NCBI Gene Expression Omnibus (GEO) under accession number GSE297990. Processed proteomics data are provided in tables in the Supporting Information, and raw mass spectrometry data are available upon reasonable request. Previously published datasets used in this study are accessible as follows: single‐nucleus RNA‐seq dataset GSE247719;^[^ ^38^ ^]^ and bulk RNA‐seq datasets GSE132040,^[^ ^18^ ^]^ GSE131901,^[^ ^19^ ^]^ GSE122080,^[^ ^53^ ^]^ and GSE247076.^[^ ^55^ ^]^ Publicly available proteomics datasets include the aging proteome dataset (https://doi.org/10.6084/m9.figshare.19765849) ^[^ ^21^ ^]^ and the longevity intervention dataset PXD040497 at http://www.ebi.ac.uk/pride.^[^ ^20^ ^]^ Other data that support the findings of this study are available from the corresponding author upon reasonable request.
