# Supplementary material for: Integrative Omics Reveal Female‐Specific Benefits of p16+ Cell Clearance in Aging Mice
Source: Adv Sci (Weinh). 2025 Oct 30;13(3):e09444. doi: 10.1002/advs.202509444 (PMC12806465; doi:10.1002/advs.202509444)
Supplement: Supplementary file 1 — Supporting Information [file ADVS-13-e09444-s002.pdf]

Supplementary Figures

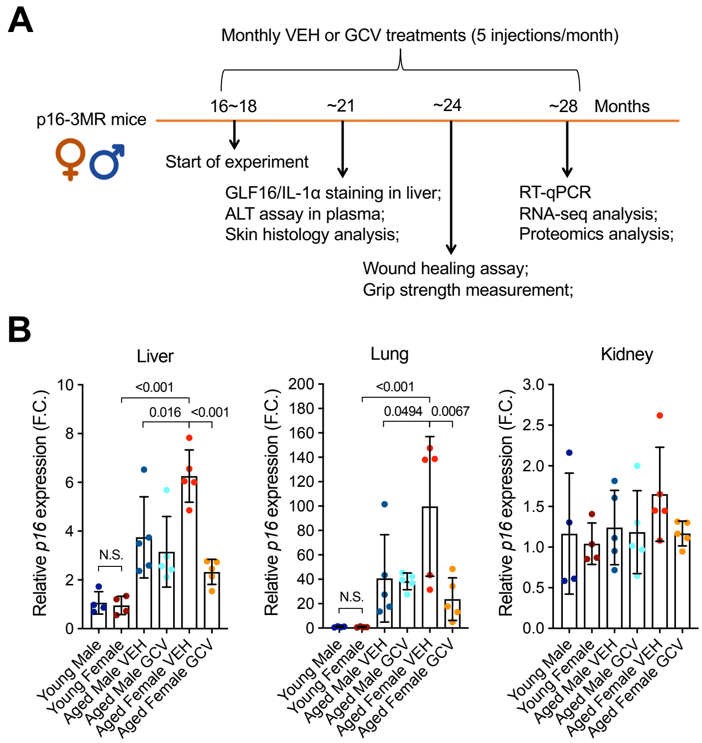

Figure S1. Differential burden and clearance responses of p16⁺ cells in aging male and female mice.

(A) Schematic experimental design for monthly VEH or GCV treatments on p16-3MR female and male mice, and follow-up analyses indicated at specific time points.

(B) RT-qPCR targeting p16 (primers targeting exon1α of mouse *Cdkn2a* transcript 2) in different groups and organs, as labelled.

For **B**, one-way ANOVA, data are shown as the mean ± SD. The p-values are labeled on the graphs. GCV, ganciclovir, was dissolved in pH11 water. VEH: vehicle (pH11 water).

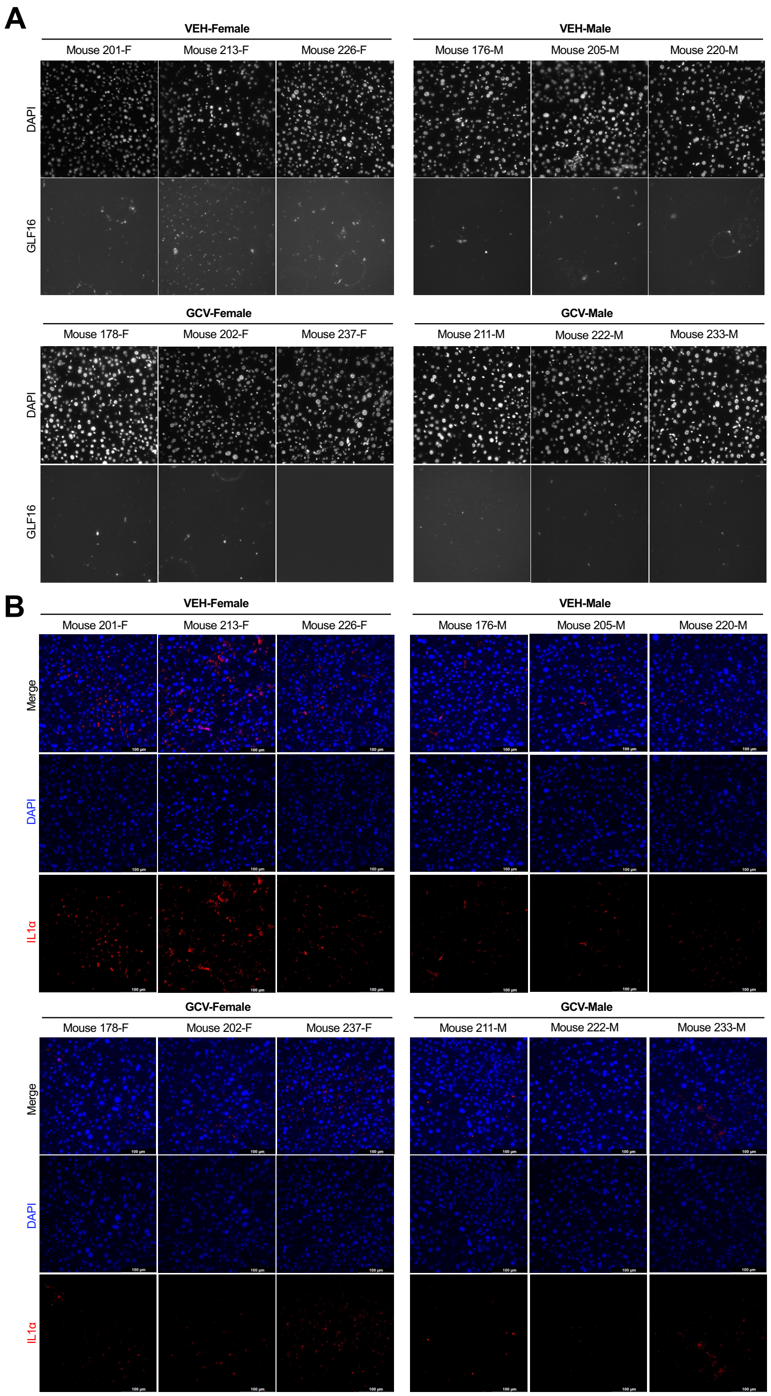

Figure S2. GCV treatments reduce p16^{+} cell burden in aged female mouse livers.
(A) Representative images with separate channels of GLF16 staining in the livers of 21-month-old VEH- or GCV-treated p16-3MR female and male mice (scale bar=100 μm, N=3 mice/group).
(B) Representative images with separate channels of IL1α staining in the livers of 21-month-old VEH- or GCV-treated p16-3MR female and male mice (scale bar=100 μm, N=3 mice/group).

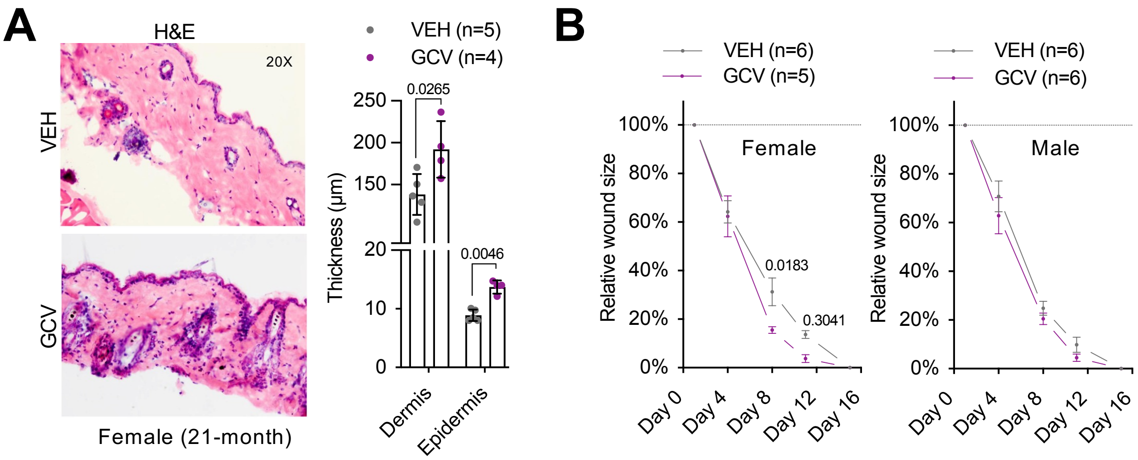

Figure S3. Clearance of p16⁺ cells improves skin health in aged female mice.
(A) Histopathology of skin biopsies from 21-month-old p16-3MR female mice treated with VEH or GCV (N=4 or n = 5 mice/group).
(B) Relative wound (4-mm punch) sizes measured over 15 days (relative to day 0) for 24-month-old p16-3MR female and male mice treated with VEH or GCV (N=5 or 6 mice/group).
For **A**, multiple *t*-test data are shown as the mean ± SD. For **B**, two-way ANOVA (Bonferroni test). Data are shown as the mean ± SEM. The p-values are labeled on the graphs. GCV, ganciclovir, was dissolved in pH11 water. VEH: vehicle (pH11 water).

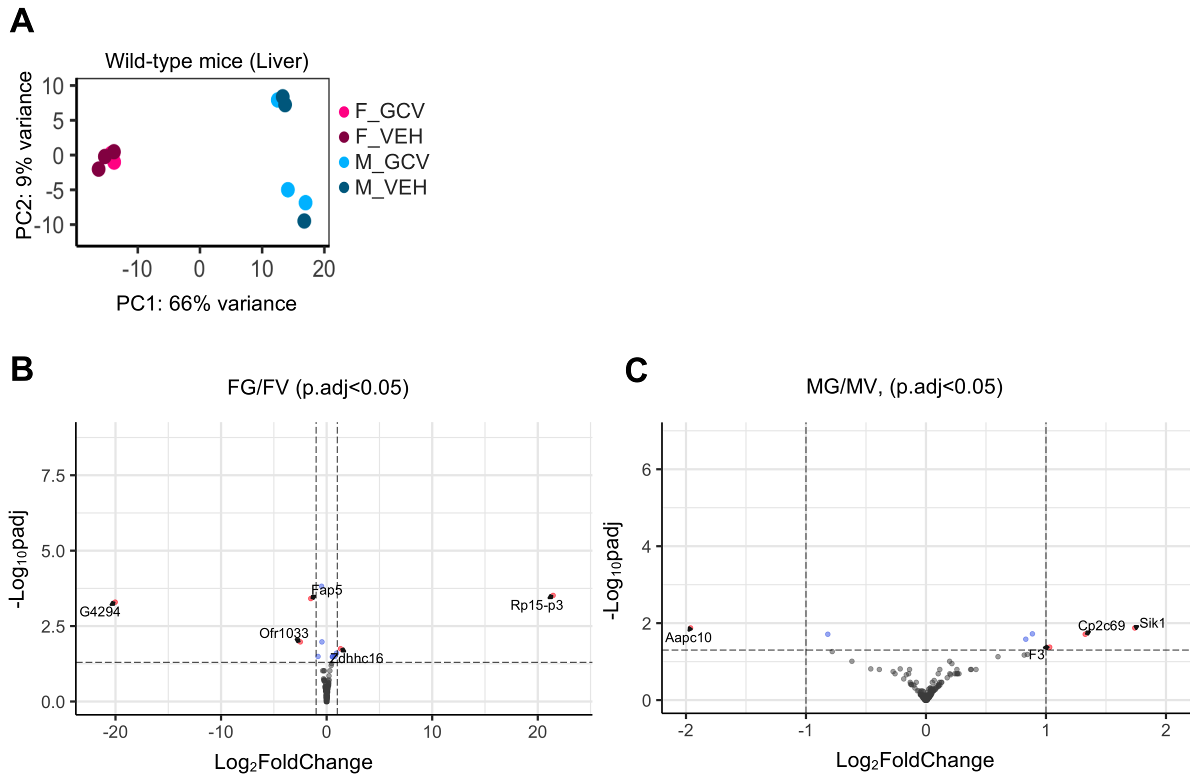

Figure S4. Transcriptional responses to GCV treatment in wild-type livers.
(A) Principal component analysis (PCA) of bulk RNA-Seq data of livers from wild-type female and male mice treated with GCV or vehicle. (N=3 per group).
(B) Volcano plots showing differentially expressed genes between GCV- and VEH- treated wild type female mice. (horizontal dashed line: p.adj = 0.05; vertical dashed line: $|\log_2 \text{FoldChange}| = 1$). (N=3 per group). Significantly altered genes are labeled with gene symbols.
(C) Volcano plots of the male mice (panel **B**).

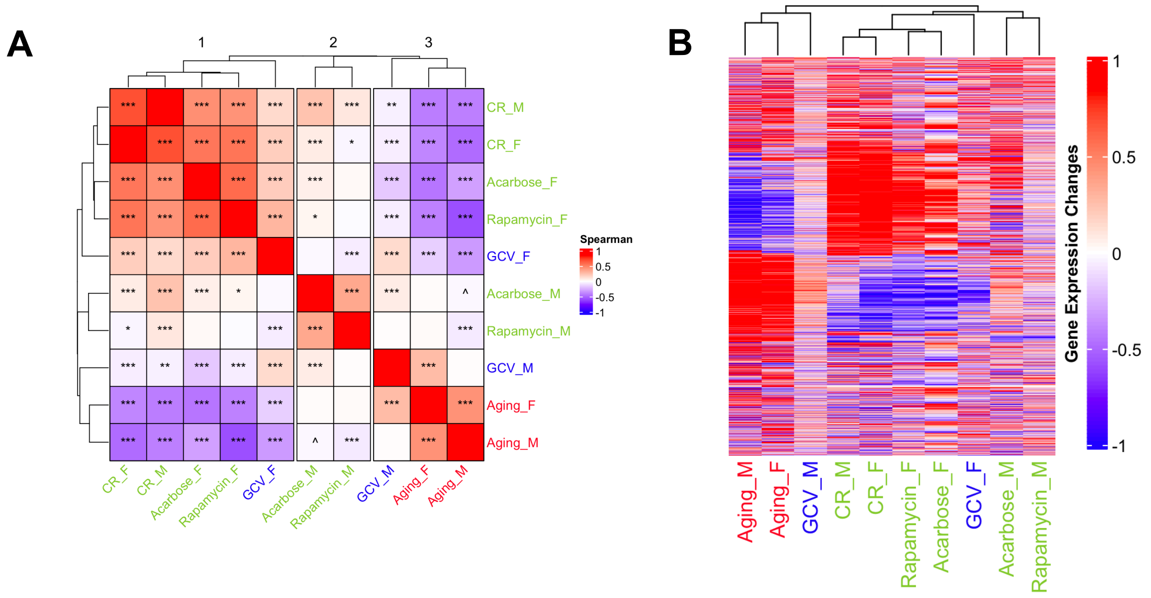

Figure S5. Gene-level transcriptomic similarity to anti-aging interventions.
(A) Hierarchical clustering and heatmap of Spearman correlations based on gene expression changes from the aging process, anti-aging interventions, and GCV treatment in both sexes. Gene expression changes were defined as $-\log_{10}(p.adj) \times \text{sign}(\log_2\text{FoldChange})$. Only genes that were significantly changed in at least one condition were included in the correlation calculation ($p.adj<0.01$, n=4,104 genes). The color intensity represents the correlation strength. Significance thresholds: ***$p<0.001$, **$p<0.01$, *$p<0.05$, ^$p<0.1$.
(B) Hierarchical clustering and heat maps showing gene expression changes as defined in **A**. Color intensity represents gene expression changes. Detailed values are listed in Table S2.

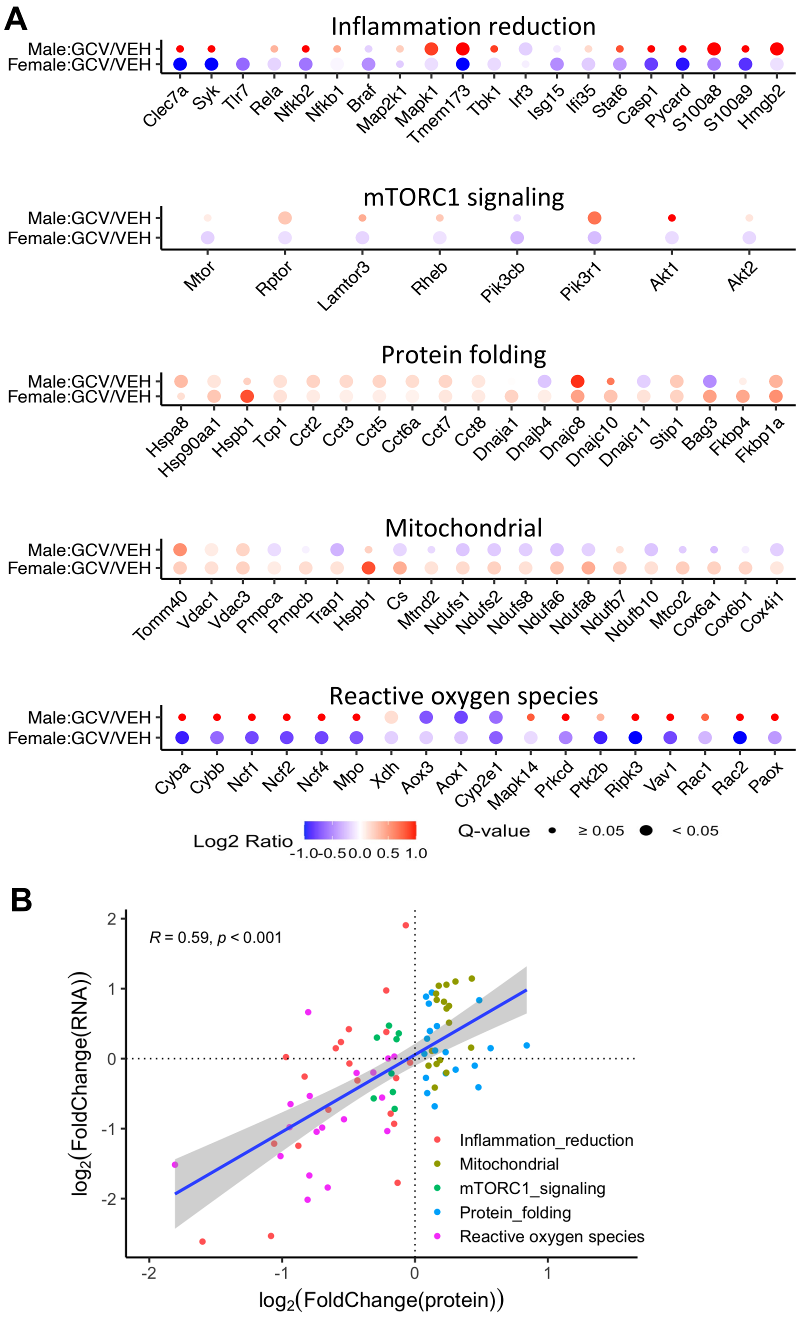

Figure S6. GCV-induced clearance of senescent cells modulates protein expression changes in rejuvenation-associated genes in 28-month-old livers.

(A) Sex-specific protein expression changes in response to GCV treatment, highlighting proteins linked to reduced inflammation, mTORC1 signaling, protein folding, mitochondrial function, and redox regulation. Point size reflects significance, and color indicates direction of change (N=3 mice/group). Detailed information for all proteins identified are listed in Table S5.

(B) Corresponding changes at both the protein and transcript levels in females for the genes shown in (A) with linear regression fit (blue line) and Spearman correlation (R=0.59, p<0.001), colored by functional category.

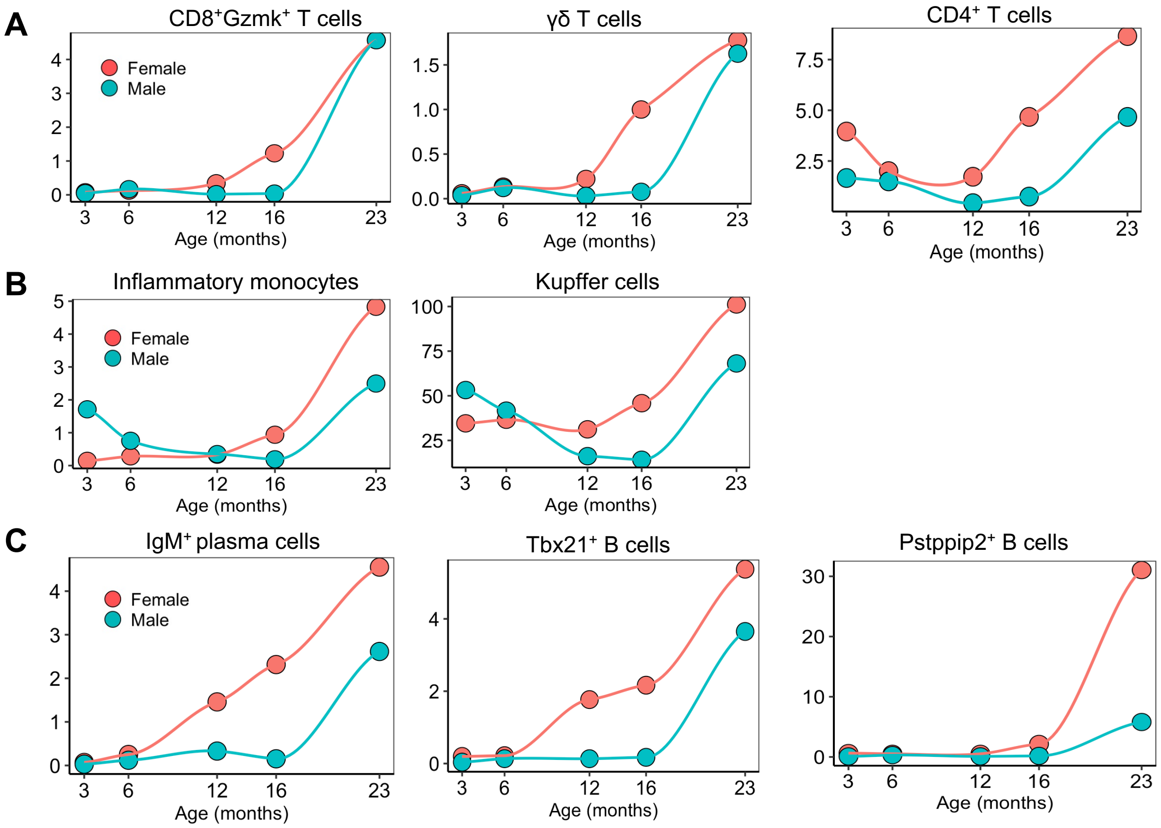

Figure S7. Age- and sex-dependent proportions of immune senescent cell types in livers.
(A) Relative abundance (per mille, ‰) of T cell subtypes, including CD8^{+}Gzmk^{+} T cells, γδ T cells, and CD4^{+} T cells, among total liver cells. Data from Zhang et al. (2025); N = 2 mice per group at 3 and 16 months, N = 4 mice per group at 6, 12, and 23 months (applies to all panels).
(B) Relative abundance of myeloid cell subtypes, including inflammatory monocytes and Kupffer cells.
(C) Relative abundance of B cell subtypes, including IgM^{+} plasma cells, Tbx21^{+} aging-associated B cells, and Pstpip2^{+} aging-associated B cells.
